# Mild-cerebellar ataxia due to impaired mitochondrial function caused by the *MSTO1* variations

**DOI:** 10.3389/fnins.2026.1775132

**Published:** 2026-04-13

**Authors:** Bin Wu, Jingwei Lv, Tong Shen, Bing Wen, Tan Wang

**Affiliations:** 1 Department of Neurology, Qilu Hospital, Shandong University, Jinan, China; 2 Department of Geriatric Medicine and Laboratory of Gerontology and Anti-Aging Research, Qilu Hospital, Shandong University, Jinan, China; 3 Department of Radiology, Qilu Hospital, Shandong University, Jinan, China

**Keywords:** cerebellar ataxia, cerebellar atrophy, mitochondrial fusion, *MSTO1*, *MSTO1*-related mitochondrial disorders

## Abstract

**Background:**

*MSTO1* encodes a regulator of mitochondrial fusion. Mutations in *MSTO1* are linked to a rare mitochondrial disorder characterized by early-onset myopathy and cerebellar ataxia, with 31 cases reported globally to date, which underscores its exceptional rarity.

**Methods:**

We conducted comprehensive clinical, molecular, and biochemical investigations in a patient harboring novel *MSTO1* variants.

**Results:**

We identified a patient presenting with adult-onset progressive ataxia and cerebellar atrophy who carried two novel compound heterozygous variants in the *MSTO1* gene (c.756A>G, p.Glu252Glu; c.1339G>A, p.Glu447Lys). Brain MRI revealed marked cerebellar abnormalities, but the patient’s clinical symptoms remained relatively mild with preserved daily function. This milder phenotype, characterized by adult onset and later disease presentation, contrasts with the more severe neurological deficits reported in a previously described case. Functional studies revealed significantly reduced MSTO1 protein expression, mtDNA depletion, and impaired mitochondrial function, as reflected by decreased mitochondrial membrane potential and respiratory capacity, suggesting a pathogenic role for these variants. Comparative analysis with fibroblasts from a previously reported case with *MSTO1* mutation revealed notable differences in the severity of mitochondrial dysfunction, suggesting potential genotype–phenotype correlations.

**Conclusion:**

Our findings provide evidence linking the novel MSTO1 variants c.756A>G and c.1339G>A to mitochondrial dysfunction and broaden the phenotypic spectrum of MSTO1-related mitochondrial disorders to encompass a milder, adult-onset form of cerebellar ataxia. These results emphasize the importance of integrated clinical and functional approaches in evaluating variant pathogenicity and in elucidating the clinical and molecular heterogeneity of *MSTO1*-related mitochondrial disorders.

## Introduction

Mitochondria are essential cytoplasmic organelles in eukaryotic cells, best known for generating adenosine triphosphate (ATP) through oxidative phosphorylation (OXPHOS) to meet cellular energy demands (Vercellino and Sazanov, 2022; Alston et al., 2021). Their proper function relies on dynamic morphology, maintained by a balance of fusion and fission processes as well as interactions with the cytoskeleton (Anesti and Scorrano, 2006). Disruption of these dynamics is increasingly implicated in various human diseases (Al Ojaimi et al., 2022).

The *MSTO1* gene (chromosome 1q22; OMIM ∗617,619) encodes the MSTO1 (Misato). Although its precise mechanistic role requires further elucidation, *MSTO1* is implicated in promoting mitochondrial fusion (Kimura and Okano, 2007; Schultz-Rogers et al., 2019). Biallelic pathogenic mutations in *MSTO1* are associated with a clinical syndrome typically featuring childhood-onset muscle weakness, hypotonia, and ataxia (Gal et al., 2017; Donkervoort et al., 2019).

Nevertheless, the complete phenotypic spectrum of *MSTO1*-related disorders and the corresponding genotype–phenotype correlations remain poorly defined. Detailed reports on the long-term clinical progression and accompanying neuroimaging evolution in patients with specific variants are notably lacking. Elucidating the natural history of these rare conditions is crucial for prognosis and management.

In this study, we described a patient with adult-onset cerebellar ataxia harboring novel compound heterozygous *MSTO1* variants (c.756A>G, p.Glu252Glu and c.1339G>A, p.Glu447Lys). Both variants were initially classified as being of uncertain significance (VUS) according to standard guidelines (Richards et al., 2015). Through a four-year longitudinal clinical assessment, progressive ataxia with correlative cerebellar atrophy on MRI was documented. We identified another patient carrying the previously reported c.727G>C (p.Ala243Pro) mutation (Liu et al., 2022), who presented with more severe symptoms and was therefore included as a positive control (PC). Cellular analyses of patient-derived fibroblasts confirmed concomitant reductions in MSTO1 protein levels and mitochondrial DNA (mtDNA) copy number. By integrating longitudinal clinical data with comprehensive functional analyses, we provide supporting evidence for the potential pathogenicity of these VUS variants and their association with mitochondrial dysfunction and associated disease. This integrative approach enhances understanding of the clinical progression and underlying pathophysiological mechanisms of *MSTO1*-related mitochondrial disorders.

## Methods

### Patient clinical presentation and follow-up study

A female patient developed an unsteady gait and experienced a fall at age 49. Her condition progressed at age 50, with frequent gait instability, dizziness, and blurred vision leading to her presentation at our hospital. The evaluation included a physical examination, cranial magnetic resonance imaging (MRI), blood biochemistry tests, liver and kidney function tests, electromyography (EMG), electroencephalography (EEG), cerebrospinal fluid (CSF) analysis, testing for demyelinating antibodies, and a muscle biopsy. To identify the cause, we performed genetic testing and investigated the health and genetic profiles of other family members. The patient was followed for 4 years, with documentation of her clinical prognosis and cranial MRI findings.

### Genetic analysis and pseudogene discrimination

Genomic DNA was extracted from peripheral blood leukocytes of the proband and available family members. Genetic investigation was performed using two complementary approaches.

First, dynamic mutation analysis was conducted to screen for common repeat expansion disorders presenting with ataxia. This included spinocerebellar ataxia (SCA) subtypes 1, 2, 3, 6, 7, 8, 10, 12, 17, 36, dentatorubral-pallidoluysian atrophy (*DRPLA*), Friedreich ataxia (*FRDA*), and fragile X syndrome (*FMR1*), using PCR and capillary electrophoresis. All results for these repeat expansions were within normal ranges. A complete list of genes analyzed by this method is provided in Supplementary Table S2.

Second, whole exome sequencing was performed using the IDT xGen Exome Research Panel v2.0 capture kit, which targets the entire human exome. This approach interrogates over 20,000 genes, including *MSTO1* and all known ataxia-associated genes. All detected variants were systematically analyzed, and a comprehensive list of candidate variants of uncertain significance (VUS) identified through this analysis, along with the rationale for their prioritization or exclusion, is provided in Supplementary Table S1. This approach ensures transparency regarding the variants considered during our study.

To specifically address the high homology with the pseudogene *MSTO2P*, capture probes were meticulously designed to target *MSTO1*-specific exonic and flanking intronic regions, thereby minimizing co-capture of pseudogene sequences.

The sequencing data were processed using a comprehensive bioinformatic pipeline as illustrated in Supplementary Figure S1. Raw reads were first merged and quality-controlled using fastp. Clean reads were then aligned to the human reference genome (GRCh38/hg38) using BWA-MEM. To ensure accurate variant calling from the *MSTO1* locus, we implemented a stringent mapping quality filter: only reads that could be uniquely mapped to *MSTO1* with high mapping quality (MAPQ ≥ 50) were retained for downstream analysis. Reads that aligned equally well to both *MSTO1* and its pseudogene *MSTO2P* were systematically excluded.

Duplicate reads were marked and removed using Picard tools. Base quality score recalibration was performed with GATK’s BaseRecalibrator, followed by variant calling using multiple algorithms including GATK HaplotypeCaller, VarDict, and Mantra. Raw variants were then recalibrated using GATK VariantRecalibrator and ApplyVQSR to filter out false positives. All candidate variants were annotated using Ensembl VEP (Variant Effect Predictor) and further prioritized with Exomiser based on phenotype compatibility and inheritance patterns.

To definitively confirm the authenticity of the reported *MSTO1* variants, Sanger sequencing was performed using primers designed to span exon-intron boundaries. This design ensures amplification from genomic DNA only, as the processed pseudogene MSTO2P lacks intronic sequences. The successful amplification and clear sequencing chromatograms provide definitive proof that these variants reside in the functional *MSTO1* gene.

A complete flowchart of this bioinformatic pipeline, including all filtering steps and quality control measures, is provided as Supplementary Figure S1. A complete list of candidate variants, including all variants of uncertain significance (VUS) identified during the analysis, along with the rationale for their prioritization or exclusion, is provided in Supplementary Table S1.

### Cell lines and culture

To investigate the functional impact of the novel compound heterozygous *MSTO1* variants (c.756A>G, p.Glu252Glu; c.1339G>A, p.Glu447Lys), primary dermal fibroblasts were cultured from the patient. Fibroblasts from a previously reported patient with pathogenic *MSTO1* variants (c.727G>C, p.Ala243Pro) served as the positive control (PC). Age-matched primary fibroblasts from a healthy individual without mitochondrial disease were used as the negative control (NC). All cell lines were maintained at 37 °C in a humidified 5% CO_2_ incubator in Dulbecco’s Modified Eagle Medium (DMEM) supplemented with 10% fetal bovine serum (FBS), 1% penicillin–streptomycin, and 1% non-essential amino acids. Fibroblasts were used between passages 4 and 8 for all experiments. Each experiment was performed using at least three independent cultures (biological replicates).

### Western blot (WB) analysis

Total protein was isolated from fibroblasts (1 × 10^6^ cells per sample). Samples were collected from three independent cultures. Proteins (20 μg) were separated by SDS-PAGE and transferred onto a PVDF membrane (Millipore, USA). After blocking with 5% skim milk for 1 h, the membrane was incubated overnight at 4 °C with primary antibodies against MSTO1 (sc-390638, SANTA) or GAPDH (ab8245, Abcam). This was followed by incubation with HRP-conjugated secondary antibodies (A0216/A0208, Beyotime). Protein bands were visualized using an ECL detection system (Tanon 4600 SF), and band intensities were quantified using ImageJ software.

### MtDNA copy number analysis

Total DNA was extracted from fibroblasts using the TIANamp Genomic DNA Kit. DNA samples were derived from three independent cultures. Relative mitochondrial DNA (mtDNA) levels were quantified by qPCR on an Applied Biosystems 7500 Real-Time PCR System using SYBR Green Master Mix (K0241, Thermo Fisher). The relative mtDNA content was normalized to nuclear 18S rRNA, with primer sequences as described in a previous publication (Eaton et al., 2007). Each sample was run in triplicate (technical replicates).

### Measurement of intracellular reactive oxygen species (DCFH-DA assay)

Cellular ROS levels were measured using the fluorescent probe DCFH-DA. Fibroblasts were incubated with 10 μM DCFH-DA at 37 °C for 30 min. For quantification, fluorescence images were captured from at least 30 randomly selected cells per condition per independent experiment (*n* = 3 independent cultures). Fluorescence was visualized by confocal microscopy, and the mean fluorescence intensity per cell was quantified using ImageJ software.

### Mitochondrial membrane potential (JC-1 assay)

Fibroblasts (1 × 10^5^ cells/well) were incubated with JC-1 dye at 37 °C for 20 min. For quantification, the red/green fluorescence ratio was determined from at least 10 randomly selected cells per condition per independent experiment (*n* = 3 independent cultures). Fluorescence (red/green ratio) was analyzed using confocal microscopy and ImageJ software.

### Mitochondrial stress test

Mitochondrial respiratory function was assessed using a Seahorse XFe24 Analyzer (Agilent). Fibroblasts from patients and controls (4 × 10^4^ cells/well) seeded in 6 replicate wells per condition (technical replicates) were analyzed in XF assay medium (pH 7.4). Data were normalized to cell count determined in parallel wells. Mitochondrial modulators were sequentially injected: 1 μM oligomycin (ATP synthase inhibitor), 1 μM FCCP (uncoupler), and 0.5 μM rotenone/antimycin A (Complex I/III inhibitors). The Oxygen Consumption Rate (OCR) was recorded at baseline and after each injection to evaluate key mitochondrial bioenergetic parameters.

### Statistical analysis

All data are derived from at least three independent and repeated experiments. For each experiment, healthy control (NC) and positive control (PC) were included for comparison. Quantitative data are presented as mean ± standard deviation (SD). Statistical comparisons were performed using one-way ANOVA, as indicated in the figure legends. Statistical significance was defined as a *p*-value ≤ 0.05. Exact *p*-values are reported in the figures or figure legends where applicable. GraphPad Prism (version 10.0) was used for generating statistical charts and for data analysis.

## Results

### Clinical presentation and genetic analysis

The patient initially developed an occasional unsteady gait at age 49. Nine months later, she was diagnosed with “cerebral infarction” and received antiplatelet, neurotrophic, and circulation-improving medications. However, her neurological symptoms continued to progress.

In 2022, she was referred to our hospital for further assessment. Neurological examination demonstrated normal muscle tone and strength in all limbs, a positive Romberg’s sign, gait ataxia, and impaired finger-to-nose and heel-to-shin tests. Autonomic function was intact. Serum creatine kinase (70 U/L), CK-MB (1.1 ng/mL), and lactate dehydrogenase (201 U/L) levels were within normal limits. Cranial MRI revealed high-signal intensities in both middle cerebellar peduncles, more prominent on the left side. Cerebrospinal fluid (CSF) analysis, including routine tests, immunoglobulin, glucose, chloride, and protein levels, was normal. Both CSF and serum tests for central nervous system demyelinating antibodies were negative, and oligoclonal bands were normal. A muscle biopsy indicated mild myopathic changes, characterized by fiber size variation with predominantly small polygonal or elongated fibers, and no evidence of necrosis or regeneration. She received symptomatic therapy with idebenone (30 mg three times daily), mecobalamin, and citicoline, but her symptoms gradually worsened.

One year later, the patient was readmitted with mild worsening of ataxia, lower limb stiffness, and clumsiness in finger movements, although she retained the ability to perform fine motor activities such as needlework. Follow-up cranial MRI showed an expanded area of abnormal signal in the right middle cerebellar peduncle, accompanied by atrophy and softening in the left middle cerebellar peduncle and left cerebellar hemisphere. Autonomic assessment revealed no significant abnormalities in supine-standing blood pressure or post-void residual urine volume (31 mL). Genetic screening for common ataxia-related genes, including spinocerebellar ataxia-associated genes and fragile X syndrome, returned normal results. Subsequent genetic analysis identified two variants in the *MSTO1* gene: c.756A>G (p.Glu252Glu) and c.1339G>A (p.Glu447Lys). Her treatment was adjusted to high-dose idebenone (150 mg twice daily), edaravone, and levocarnitine, which she tolerated well. She reported subjective improvements in balance and reduced fear of walking.

**Figure 1:**
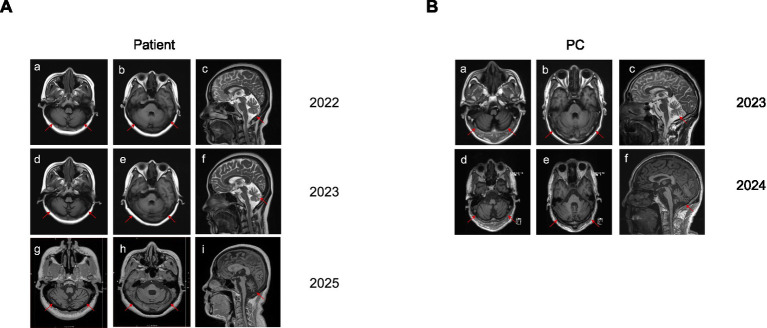
Serial cranial MRI demonstrating progressive cerebellar atrophy in the patient. **(A)** Serial cranial MRI scans of the patient obtained over a four-year follow-up period (2022, 2023, and 2025). (**A**, a–c) Baseline scan (2022). (**A**, d–f) One-year follow-up scan (2023). (**A**, g–i) Three-year follow-up scan (2025). **(B)** Serial cranial MRI scans of the PC obtained over a two-year follow-up period (2023 and 2024). (**B**, a–c) Baseline scan (2023). (**B**, d–f) One-year follow-up scan (2024). The arrow indicates the area of cerebellar atrophy.

Over a four-year follow-up period, the patient’s ataxia symptoms remained stable without significant progression. In contrast, the previously reported case carrying *MSTO1* mutations presented with a much more severe phenotype, exhibiting near-total loss of autonomous mobility. This highlights the relatively mild clinical course in our patient. Blood pressure and post-void residual urine volume remained normal. Cranial MRI scans obtained during all three admissions are presented, including axial views of the cerebellar hemispheres and vermis, as well as coronal sections (Figure 1).

### Pedigree analysis

**Figure 2:**
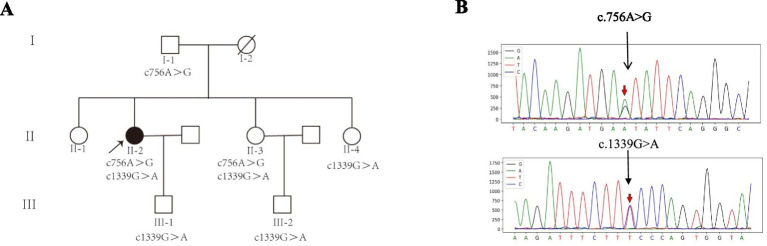
Pedigree, genetic variants of the patient. **(A)** Pedigree of the family. The proband (patient, indicated by an arrow) and her younger sister (II-3) are compound heterozygotes for the *MSTO1* variants. Family members carrying a single variant are unaffected carriers. The genotype for each available individual is indicated below the symbol. **(B)** Sanger sequencing chromatograms confirming the heterozygous *MSTO1* variants c.756A>G and c.1339G>A in the proband (patient). The arrow indicates the mutation site.

The patient and her younger sister were both compound heterozygotes for the *MSTO1* variants, having inherited the c.756A>G (p.Glu252Glu) from their father and the c.1339G>A (p.Glu447Lys) from their mother (Figures 2A,B). The younger sister, who currently exhibits a mild unsteady gait, could not be thoroughly evaluated or followed up due to lack of family cooperation, thus limiting the available clinical data. The father, another sister, and the patient’s son, all carriers of a single heterozygous variant, report no neurological symptoms and have not undergone detailed clinical evaluation. This co-segregation of the variants with the disease phenotype within the family supports a potential pathogenic role for both *MSTO1* c.756A>G and c.1339G>A. Although c.756A>G is a synonymous variant, its segregation with the disease and the observed reduction in MSTO1 protein suggest a potential functional impact, possibly through mechanisms such as effects on mRNA stability or translational efficiency, which merit further investigation.

### MSTO1 protein expression is reduced in patient fibroblasts

**Figure 3:**
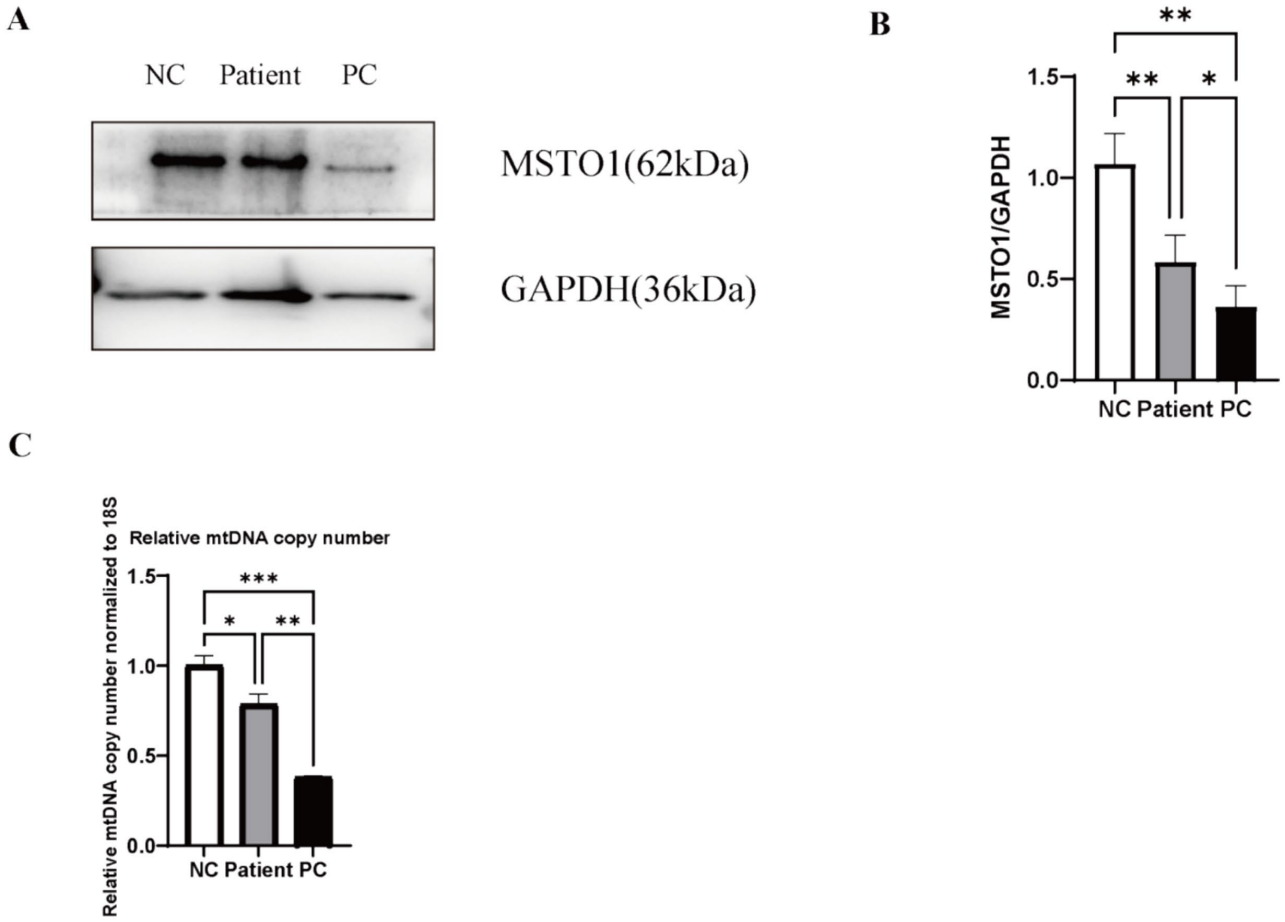
Reduced MSTO1 protein expression and mitochondrial DNA (mtDNA) depletion in patient fibroblasts. **(A)** Representative immunoblot of MSTO1 protein in fibroblast lysates from the NC, the patient, and the PC. GAPDH served as the loading control. **(B)** Densitometric quantification of MSTO1 protein levels normalized to GAPDH. (Data from *n* = 3 independent experiments; one-way ANOVA, ****p < 0.0001 vs. NC, ****p < 0.001 vs. PC). **(C)** Quantitative PCR analysis of the mitochondrial DNA to nuclear DNA (mtDNA/nDNA) ratio. Fibroblasts from patient and PC show significant mtDNA depletion compared to the NC. (Data from *n* = 3 independent experiments, each run in triplicate; one-way ANOVA, *p < 0.05 vs. NC, *p < 0.05 vs. PC).

Immunoblot analysis of fibroblast lysates revealed a significant decrease in MSTO1 protein levels in patient compared to the NC, a finding confirmed by densitometric quantification. Further analysis demonstrated that this reduction was less pronounced in patient than in the PC (Figures 3A,B), indicating an intermediate level of protein loss.

### Patient fibroblasts exhibit mtDNA depletion

Quantitative PCR analysis revealed a marked reduction in the mitochondrial DNA (mtDNA) copy number in fibroblasts from the patient and PC. The mtDNA depletion was less pronounced in patient than in the PC (Figure 3C). This observed depletion provides a key molecular link between the *MSTO1* variants and the downstream functional impairments in mitochondrial function.

### Patient fibroblasts display elevated intracellular ROS levels

**Figure 4:**
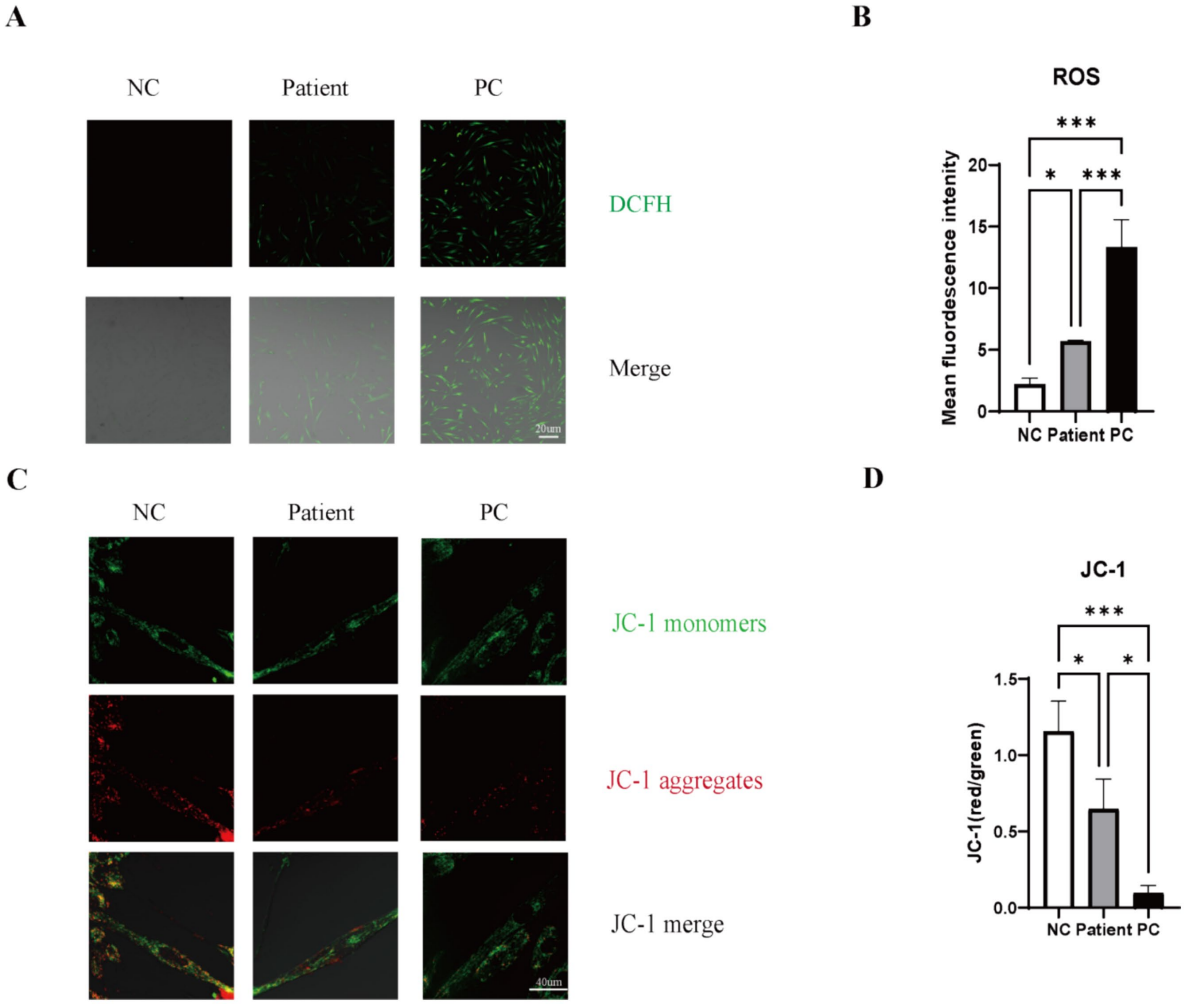
Assessment of mitochondrial dysfunction and oxidative stress in patient fibroblasts. **(A)** Representative confocal microscopy images showing DCFH-DA fluorescence (green) in NC, patient, and PC fibroblasts. Scale bar: 20 μm. **(B)** Quantitative analysis of fluorescence intensity revealed a significant increase in ROS levels in both patient and PC cells compared to the NC. The increase was more pronounced in PC cells than in patient cells. Data are presented as mean ± SD (*n* ≥ 30 cells from 3 independent cultures; one-way ANOVA, *p < 0.05 vs. NC, ***p < 0.001 patient vs. PC). **(C)** Representative confocal microscopy images of JC-1 staining. Red fluorescence (J-aggregates) indicates high ΔΨm, while green fluorescence (J-monomers) indicates low ΔΨm. Scale bar: 40 μμm. **(D)** Quantitative analysis of the red/green fluorescence ratio showed a significant decrease in ΔΨm in both patient and PC cells compared to the NC, indicating mitochondrial depolarization. The depolarization in patient cells was intermediate between the NC and the severely affected PC cells. Data are presented as mean ± SD (*n* ≥ 10 cells from 3 independent cultures; one-way ANOVA, *p < 0.05 vs. NC, *p < 0.05 vs. PC, ***p < 0.001 PC vs. NC).

Confocal microscopy imaging of DCFH-DA-stained fibroblasts revealed a marked increase in green fluorescence in patient cells compared to the healthy control, indicating elevated levels of intracellular reactive oxygen species (ROS). Quantitative analysis confirmed a statistically significant increase in ROS in both patient and PC fibroblasts. Notably, the ROS level in PC cells was significantly higher than that in patient (Figures 4A,B). This finding demonstrates that the *MSTO1* variants are associated with significant oxidative stress.

### Patient fibroblasts exhibit partial loss of mitochondrial membrane potential

Confocal microscopy imaging of JC-1-stained fibroblasts revealed a clear reduction in the red/green fluorescence ratio in the patient’s cells compared to the healthy control, indicating a loss of mitochondrial membrane potential (ΔΨm). Quantitative analysis using ImageJ software confirmed a statistically significant decrease in the patient’s ΔΨm (Figures 4C,D). However, this depolarization was less severe than that observed in the positive control cells, which carried known pathogenic *MSTO1* mutations and exhibited near-complete mitochondrial depolarization. These results demonstrate that the novel *MSTO1* variants in our patient are associated with a partial, yet significant, impairment of mitochondrial function.

### Severe impairment of mitochondrial respiratory function

To comprehensively assess the bioenergetic consequences, mitochondrial respiration was analyzed using the Seahorse XF Analyzer. The OCR trajectory of patient fibroblasts was significantly lower than that of NC cells throughout the assay.

**Figure 5:**
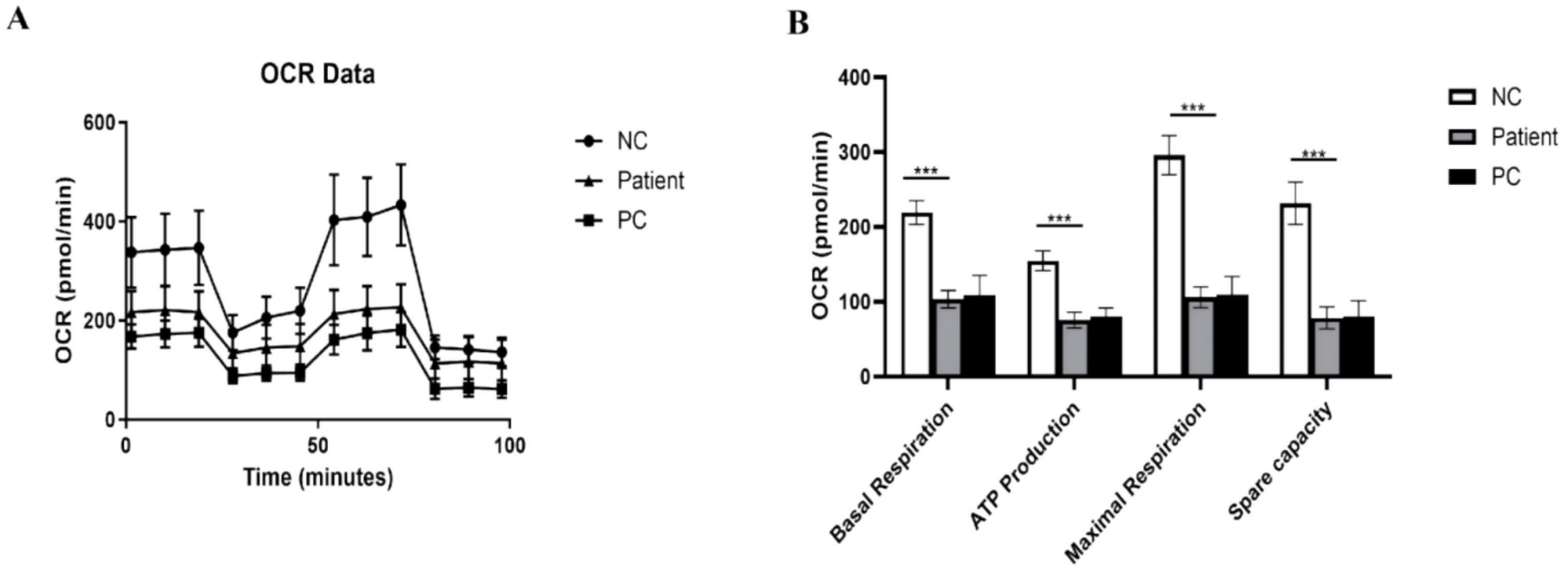
Functional assessment reveals impaired respiratory capacity. **(A)** Mitochondrial respiratory function analyzed by Seahorse XF cell mito stress test. The oxygen consumption rate (OCR) profile in response to oligomycin, FCCP, and rotenone/antimycin A is shown. **(B)** Quantification of key respiratory parameters: basal respiration, ATP-linked respiration, maximal respiration, and spare respiratory capacity (data normalized to cell count, presented as mean ± SD from *n* = 3 independent experiments, each with 6 technical replicates; one-way ANOVA, ***p < 0.001 vs. NC).

Key parameters derived from the OCR data were quantified. Basal respiration was significantly impaired in patient cells. More strikingly, the maximum respiratory capacity, induced by the uncoupler FCCP, was severely compromised. Consequently, ATP-linked respiration was also markedly reduced (Figures 5A,B). These data demonstrate profound and widespread defects in mitochondrial oxidative phosphorylation. Notably, patient cells exhibited a similar trend of respiratory impairment compared to the PC cells, with no significant difference in the severity of these defects.

### ACMG-based variant classification

To systematically evaluate the pathogenicity of the two *MSTO1* variants identified in our patient, we applied the ACMG/AMP guidelines (Richards et al., 2015). The synonymous variant c.756A>G (p.Glu252Glu) is rare in population databases, with an allele frequency of approximately 0.00033 in the East Asian population of gnomAD and no reported homozygotes, satisfying criterion PM2_supporting. In silico splice prediction tools do not predict an effect on splicing. It also co-segregates with the disease phenotype in the family, meeting criterion PP1_supporting. Most importantly, our functional studies demonstrated significantly reduced MSTO1 protein expression, mtDNA depletion, decreased mitochondrial membrane potential, and impaired respiratory capacity, providing moderate evidence of pathogenicity (PS3_Moderate). Based on these combined criteria, and considering the most recent ClinGen guidance which recommends PM2_supporting at supporting strength only, the cumulative evidence supports a classification of Variant of Uncertain Significance (VUS) for c.756A>G.

For the missense variant c.1339G>A (p.Glu447Lys), it is also rare in population databases (PM2_supporting) and shows co-segregation with disease (PP1_supporting). Multiple in silico predictors give conflicting results, with most predicting a tolerated effect; therefore, PP3 is not applied. Functional evidence again supports a damaging effect (PS3_Moderate). Similarly, c.1339G>A is also classified as Variant of Uncertain Significance (VUS).

## Discussion

Our study identifies significant mitochondrial failure, characterized by diminished membrane potential and impaired respiratory function, in patient-derived fibroblasts harboring compound heterozygous *MSTO1* mutations. While previous studies on severe, early-onset *MSTO1*-related myopathy (Schultz-Rogers et al., 2019; Ardicli et al., 2019; Nasca et al., 2021; Chen et al., 2022; Iwama et al., 2018) have established a clear link to mitochondrial dysfunction (Nasca et al., 2017), our findings reveal that the specific mutations in our patient are associated with a quantitatively milder functional impairment. This was evidenced by a direct comparison with fibroblasts from a patient (PC) carrying a known severe pathogenic mutation, in which our patient exhibited less severe reductions in protein expression, mtDNA copy number. We thus hypothesize that the spectrum of *MSTO1*-related mitochondrial disorders may be defined by a genotype–phenotype correlation, where the severity of the molecular defect dictates the clinical outcome.

The relatively mild muscle histopathology and normal serum markers in our patient contrast with the severe myopathic changes often described in early-onset *MSTO1* cases (Schultz-Rogers et al., 2019; Liu et al., 2022; Nasca et al., 2017). This discrepancy likely reflects the phenotypic heterogeneity of *MSTO1*-related disorders. Tissue-specific vulnerability may play a role, with cerebellar neurons being particularly sensitive to mitochondrial dysfunction, while skeletal muscle remains relatively spared, especially in adult-onset forms. Alternatively, our patient may have been biopsied at an early stage before significant muscle pathology developed. Similar observations have been reported in other mitochondrial disorders where neurological symptoms precede overt myopathy The relatively preserved mitochondrial function in patient could explain the later onset and the predominantly cerebellar, rather than muscular, involvement in our case. This model provides a potential compelling cellular basis for the patient’s specific phenotype, which likely stems from a slower, more selective neuronal energy deficit over time.

We provide functional evidence supporting a role for the *MSTO1* c.756A>G and c.1339G>A variants in mitochondrial dysfunction. A comparative analysis with fibroblasts from a patient carrying a known pathogenic mutation (PC) revealed that our patient exhibited a milder impairment at the molecular and functional levels, including protein expression, mtDNA copy number, and respiratory capacity. This graded severity is consistent with the possibility that the c.756A>G synonymous variant might have a less detrimental impact on mitochondrial function than the missense variant carried by PC. The presence of only mild symptoms in the genotypically identical sister further supports this genotype–phenotype correlation, possibly representing an earlier stage of the same disease spectrum.

Although the patient’s progressive cerebellar ataxia and specific MRI findings initially suggested multiple system atrophy (MSA), several key observations argue against this diagnosis. The clinical presentation diverged from the typical profile of MSA. Notably, the patient lacked the progressive and severe autonomic failure core to current diagnostic criteria (Gilman et al., 2008; Fanciulli and Wenning, 2021), with repeated assessments showing no significant orthostatic hypotension and only stable, mild residual urine volume (Oertel et al., 2003). Furthermore, no parkinsonian signs (Osaki et al., 2009) were observed throughout the disease course. In addition, the clinical course has remained notably stable over 4 years of follow-up, in sharp contrast to the rapid deterioration characteristic of MSA. Finally, the identification of biallelic *MSTO1* mutations provides a definitive genetic explanation consistent with a hereditary mitochondrial ataxia, offering a specific alternative diagnosis that aligns with the clinical and laboratory findings.

This study is limited by its focus on a single family, which restricts the generalizability of the phenotypic spectrum associated with the novel *MSTO1* variants. Although the younger sister of the patient shares the identical genotype and exhibits early signs of disease, a comprehensive longitudinal follow-up was not possible due to lack of family consent. This prevented a deeper analysis of disease progression and penetrance within this specific genetic context. Furthermore, the use of a fibroblast model may not fully capture the tissue-specific pathophysiology, particularly in the cerebellum. Moreover, while our experimental data connect the variants to a defined set of mitochondrial impairments such as bioenergetic failure, mtDNA depletion, and elevated oxidative stress, the precise molecular mechanism underlying the loss of MSTO1 protein function remains unresolved. Specifically, further RNA-level studies are warranted to elucidate the molecular mechanism by which this disease-associated synonymous variant c.756A>G exerts its functional effect. Synonymous variants can influence gene expression and contribute to disease through diverse mechanisms. These include potential effects on mRNA splicing, stability, translational efficiency, or transcriptional regulation. While our functional data link the *MSTO1* c.756A>G variant to reduced protein levels, the precise molecular pathway requires further elucidation. Future studies involving transcript quantification, analysis of RNA secondary structure, and assessment of ribosomal engagement will be essential to determine the mechanism by which this synonymous variant exerts its effect. The exact mechanism by which these mutations impair *MSTO1* function, and their downstream consequences on mitochondrial dynamics, warrants further investigation. We acknowledge that according to current ACMG guidelines, the cumulative evidence supports a classification of Variant of Uncertain Significance (VUS) for both variants. This classification reflects the limited number of reported cases, the supporting-strength application of PM2_supporting, and the lack of transcript-level analyses. Nevertheless, the consistent and severe mitochondrial dysfunction observed in patient-derived fibroblasts, together with the clear clinical phenotype and family segregation, strongly supports a causative role for these variants. As more *MSTO1* cases are reported, the weight of evidence may increase, potentially leading to reclassification as “Pathogenic.” Future studies employing isogenic cellular models or gene-edited animals will be crucial to delineate the causal mechanisms and explore therapeutic avenues.

Finally, given the rarity of this disorder, the phenotypic and functional observations presented here should be interpreted as describing this specific familial context; broader generalizations await confirmation in additional, independent cases.

## Conclusion

In summary, our integrated clinical and functional study provides compelling evidence that the novel compound heterozygous *MSTO1* variants (c.756A>G, p.Glu252Glu; c.1339G&A, p.Glu447Lys) are associated with mitochondrial dysfunction and a distinct clinical phenotype. We have expanded the phenotypic spectrum of *MSTO1*-related mitochondrial disorders by characterizing a mild, adult-onset, cerebellar ataxia presentation, which stands in contrast to more severe, early-onset neurodevelopmental forms. Through a four-year longitudinal follow-up, we documented the slowly progressive yet relatively mild nature of this disease variant, which contrasts with the more severe early-onset forms. Cellular analyses confirmed mitochondrial dysfunction, demonstrating a distinctive pattern where significant respiratory deficiency coexists with partial impairment in other parameters. Crucially, our comparative functional approach established measurable differences in severity between patient cell lines, contributing to our understanding of genotype–phenotype correlations in *MSTO1*-related mitochondrial disorders and providing insights valuable for both diagnosis and prognostic assessment.

## Data Availability

The original contributions presented in the study are included in the article/Supplementary material, further inquiries can be directed to the corresponding author.
