# Supplementary figures and images for: Mild-cerebellar ataxia due to impaired mitochondrial function caused by the MSTO1 variations
Source: Front Neurosci. 2026 Apr 13;20:1775132. doi: 10.3389/fnins.2026.1775132 (PMC13111549; doi:10.3389/fnins.2026.1775132)

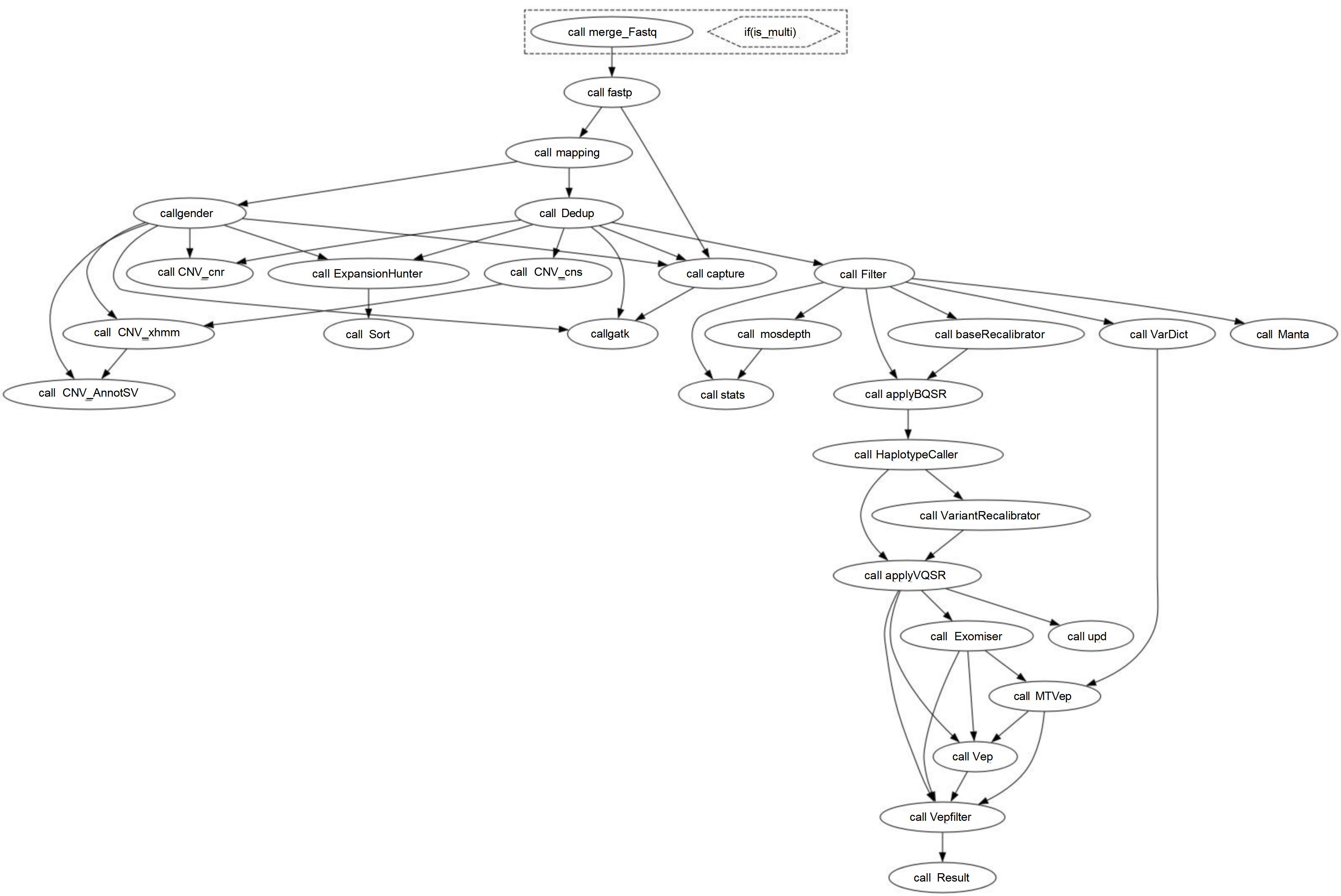

Supplement: Supplementary file 1 [file Image_1.png]
