# Supplementary material for: Mild-cerebellar ataxia due to impaired mitochondrial function caused by the MSTO1 variations
Source: Front Neurosci. 2026 Apr 13;20:1775132. doi: 10.3389/fnins.2026.1775132 (PMC13111549; doi:10.3389/fnins.2026.1775132)
Supplement: Supplementary file 2 [file Table_1.docx]

Supplementary Table S1. List of candidate variants of uncertain significance (VUS) identified in other genes and rationale for exclusion

| **Gene** | **Chromosome position**  **(hg19)** | **Transcript** | **Nucleotide change** | **Amino acid change** | **Zygosity** | **Population frequency**  **(gnomAD East Asian)** | **Inheritance pattern**  **(associated disease)** |
| --- | --- | --- | --- | --- | --- | --- | --- |
| *HGD* | chr3:120352019 | NM_000187;  exon13 | c. 1163C>T | p.Pro388Leu | Heterozygous | 0.000054 | AR(Alkaptonuria,OMIM(203500) |
| *SYNE1* | chr6:152751312 | NM_033071;  exon36 | c.4744C>T | p.Leu1582Phe | Heterozygous | 0.006391 | AR (SCAR8, OMIM 610743); AD (EDMD4, OMIM 612998)） |
| *PCLO* | chr7:82595447 | NM_033026;  exon4 | c.3657A>T | p.Lys1219Asn | Heterozygous | 0 | AR (PCH3, OMIM 608027) |
| *FRMD4A* | chr10:13699364 | NM_001318  337;exon21 | c.2324A>G | p.Asp775Gly | Heterozygous | 0 | AR(Corpus callosum hypoplasia with ataxia, OMIM 616819) |
| *TWNK* | chr10:102749108 | NM_021830;  exon1 | c. 1141C>G | p.Leu381Val | Heterozygous | 0 | AR (Perrault syndrome 5, OMIM 616138; mtDNA depletion syndrome 7, OMIM 271245); AD (PEOA3, OMIM 609286) |
| *TWNK* | chr10:102749163 | NM_021830;  exon1 | c. 1196A>G | p.Asn399Ser | Heterozygous | 0.000054 | Same as above |
| *HTRA1* | chr10:124248990 | NM_002775;  exon3 | c.625G>C | p.Val209Leu | Heterozygous | 0 | AD (CADASIL2, OMIM 616779); AR (CARASIL, OMIM 600142)） |
| *SPTBN2* | *chr11:66472288* | *NM_006946;exon14* | *c.2459C>T* | *p.Thr820Met* | *Heterozygous* | *0.002099* | AD (SCA5, OMIM 600224); AR (SCAR14, OMIM 615386) |
| *COL4A1* | *chr13:110802732* | *NM_001845;*  *exon52* | *c.4988A>G* | *p.Gln1663Arg* | *Heterozygous* | *0* | AD (Brain small vessel disease with or without ocular anomalies, OMIM 175780; etc.) |
| *NDUFAF1* | *chr15:41679648* | *NM_016013;*  *exon5* | *c.978T>A* | *p.Phe326Leu* | *Heterozygous* | *00.000109* | AR (Mitochondrial complex I deficiency, OMIM 252010; nuclear type 11, OMIM 618234) |
| *BBS1* | chr11:66282015 | NM_024649;  exon4 | c.298C>T | p.Arg100Trp | Heterozygous | 0 | DR/AR (Bardet-Biedl syndrome 1, OMIM 209900) |

All variants listed are classified as Variants of Uncertain Significance (VUS) according to ACMG guidelines. The rationale for exclusion is based on inheritance pattern incompatibility (single heterozygosity for AR disorders), lack of phenotype matching, relatively high population frequency, and/or absence of functional evidence supporting pathogenicity. The two MSTO1 variants reported in this study (c.756A>G and c.1339G>A) were prioritized due to their biallelic state, rarity, consistent phenotype with MSTO1-related mitochondrial disorder (cerebellar ataxia with mitochondrial dysfunction), and functional evidence demonstrating impaired MSTO1 protein expression and mitochondrial bioenergetics.
