# Supplementary material for: Mild-cerebellar ataxia due to impaired mitochondrial function caused by the MSTO1 variations
Source: Front Neurosci. 2026 Apr 13;20:1775132. doi: 10.3389/fnins.2026.1775132 (PMC13111549; doi:10.3389/fnins.2026.1775132)
Supplement: Supplementary file 3 [file Table_2.docx]

Supplementary Table S2. Genes analyzed by dynamic mutation analysis for repeat expansion disorders.

| **Gene** | **Associated Disease** | **Inheritance Pattern** | **OMIM ID** | **Result** |
| --- | --- | --- | --- | --- |
| ATXN1 | Spinocerebellar ataxia type 1 (SCA1) | AD | 164400 | Normal |
| ATXN2 | Spinocerebellar ataxia type 2 (SCA2) | AD | 183090 | Normal |
| ATXN3 | Spinocerebellar ataxia type 3 (SCA3) | AD | 109150 | Normal |
| CACNA1A | Spinocerebellar ataxia type 6 (SCA6) | AD | 183086 | Normal |
| ATXN7 | Spinocerebellar ataxia type 7 (SCA7) | AD | 164500 | Normal |
| ATXN8OS/ATXN8 | Spinocerebellar ataxia type 8 (SCA8) | AD | 608768 | Normal |
| ATXN10 | Spinocerebellar ataxia type 10 (SCA10) | AD | 603516 | Normal |
| PPP2R2B | Spinocerebellar ataxia type 12 (SCA12) | AD | 604326 | Normal |
| TBP | Spinocerebellar ataxia type 17 (SCA17) | AD | 600075 | Normal |
| NOP56 | Spinocerebellar ataxia type 36 (SCA36) | AD | 614154 | Normal |
| ATN1 | Dentatorubral-pallidoluysian atrophy (DRPLA) | AD | 607462 | Normal |
